# Hypoxia and proangiogenic proteins in human ameloblastoma

**DOI:** 10.1038/s41598-020-74693-7

**Published:** 2020-10-16

**Authors:** Raíssa Pinheiro de Mendonça, Karolyny Martins Balbinot, Beatriz Voss Martins, Maria Sueli da Silva Kataoka, Ricardo Alves Mesquita, João de Jesus Viana Pinheiro, Sérgio de Melo Alves Júnior

**Affiliations:** 1 Department of Oral Pathology, School of Dentistry, Universidade Federal do Pará, Avenida Augusto Correa, 01, Belém, Pará 66075-110 Brazil; 2 Department of Oral Surgery and Pathology, School of Dentistry, Universidade Federal de Minas Gerais, Avenida Presidente Antônio Carlos, 6627, Belo Horizonte, Minas Gerais 31270-901 Brazil

**Keywords:** Oncology, Pathogenesis, Medical research, Biomarkers, Cell biology, Mechanisms of disease, Biomarkers, Prognostic markers, Diseases, Dental diseases

## Abstract

Ameloblastomas are epithelial odontogenic tumours that, although benign, are locally invasive and may exhibit aggressive behaviour. In the tumour microenvironment, the concentration of oxygen is reduced, which leads to intratumoral hypoxia. Under hypoxia, the crosstalk between the HIF-1α, MMP-2, VEGF, and VEGFR-2 proteins has been associated with hypoxia-induced angiogenesis, leading to tumour progression and increased invasiveness. This work showcases 24 ameloblastoma cases, 10 calcifying odontogenic cysts, and 9 dental follicles, used to investigate the expression of these proteins by immunohistochemistry. The anti-HIF-1α, anti-MMP-2, anti-VEGF, and anti-VEGFR-2 primary antibodies are used in this work. The results have been expressed by the mean grey value after immunostaining in images acquired with an objective of 40×. The ameloblastoma samples showed higher immunoexpression of HIF-1α, MMP-2, VEGF, and VEGFR-2 when compared to the dental follicles and calcifying odontogenic cysts. Ameloblastomas show a higher degree of expression of proteins associated with intratumoral hypoxia and proangiogenic proteins, which indicates the possible role of these proteins in the biological behaviour of this tumour.

## Introduction

Odontogenic cysts and tumours comprise a large part of oral and maxillofacial pathologies, and, therefore, there is a need to explore their biological behaviour, deepening knowledge about their development and progression.

Ameloblastomas (AMEs) are one of the most common odontogenic tumours. They are characterized by a slow growth, high recurrence and morbidity rates, and local invasiveness^1^. Although surgery is the most acceptable treatment modality so far, it produces severe aesthetic and functional sequelae that lead to a loss of life quality^2^.

A calcifying odontogenic cyst (COC) is an odontogenic cystic lesion that has a less aggressive behaviour than an AME. The prognosis of a patient with COC is favourable, with few recurrences reported after simple enucleation^1,3^.

During tumour progression, the concentration of oxygen in the microenvironment around the tumour cells is reduced, resulting in intratumoral hypoxia, characterized by reduced oxygen pressure in the cells, which leads to various biochemical responses and may result in a number of compensatory cellular mechanisms that allow the continuation of neoplastic development. A hypoxic microenvironment is characteristic of many solid tumours. Hypoxia is also associated with a more aggressive phenotype, which affects angiogenesis and cellular invasiveness^4,5^.

Angiogenesis and cellular invasion are complex multistage processes that begin with extracellular matrix (ECM) degradation caused by proteolytic enzymes, matrix metalloproteinases (MMPs), and, especially, cancer-associated MMP-2 and -9^6–9^, which stimulate the release of angiogenic and growth factors in the basement membrane, such as the vascular endothelial growth factor (VEGF/VEGFA)^10^.

The signalling pathway involving VEGF/VEGFA and VEGFR is a promising target for cancer treatment, as it has been identified as the main regulator of tumour angiogenesis^11–15^. Among VEGF receptors (VEGFRs), VEGFR-2 is overexpressed under hypoxia^15^. VEGFR-2 plays an important role in activating the components responsible for proliferation, including endothelial cell invasion, migration, differentiation, and angiogenesis^16^.

Some of these proteins have already been studied in AME, COC and DF. Three studies verified the expression of HIF-1 α and found a higher expression of this protein in AME when compared to COC and DF, and suggested that may be associated with its aggressive biological behaviour^17–19^. Three studies have verified the expression of VEGF and found a higher expression of this protein in AME when compared to odontogenic keratocyst, dentigerous cyst or tooth germ, suggesting that the up-regulation of this protein in AME might be associated with neoplastic or malignant changes of odontogenic epithelial cells^20–22^. There are no studies on the expression of VEGFR-2 in these pathologies or on the relationship between all these proteins.

Studies in skin, breast, gastric, brain, salivary gland, and pulmonary cancer have shown the role of these proteins in promoting tumour progression, where the hypoxia-inducible factor 1 alpha (HIF-1α) plays a regulatory role. When HIF-1α expression is stabilized, through hypoxia, it can induce MMP-2 expression, which degrades ECM, stimulating the expression of growth factors such as VEGF, which binds to its receptor VEGFR-2, initiating a series of processes, including angiogenesis, proliferation, and invasion, which will contribute to tumour growth^23–31^. In addition, HIF-1 may act as a direct transcriptional activator for the VEGF gene promoter^32,33^.

Therefore, the objective of this study was to analyse the expression of these proteins in AME, as this biological approach may be useful for better understanding the behaviour of this neoplasm.

## Results

**Figure 1:**
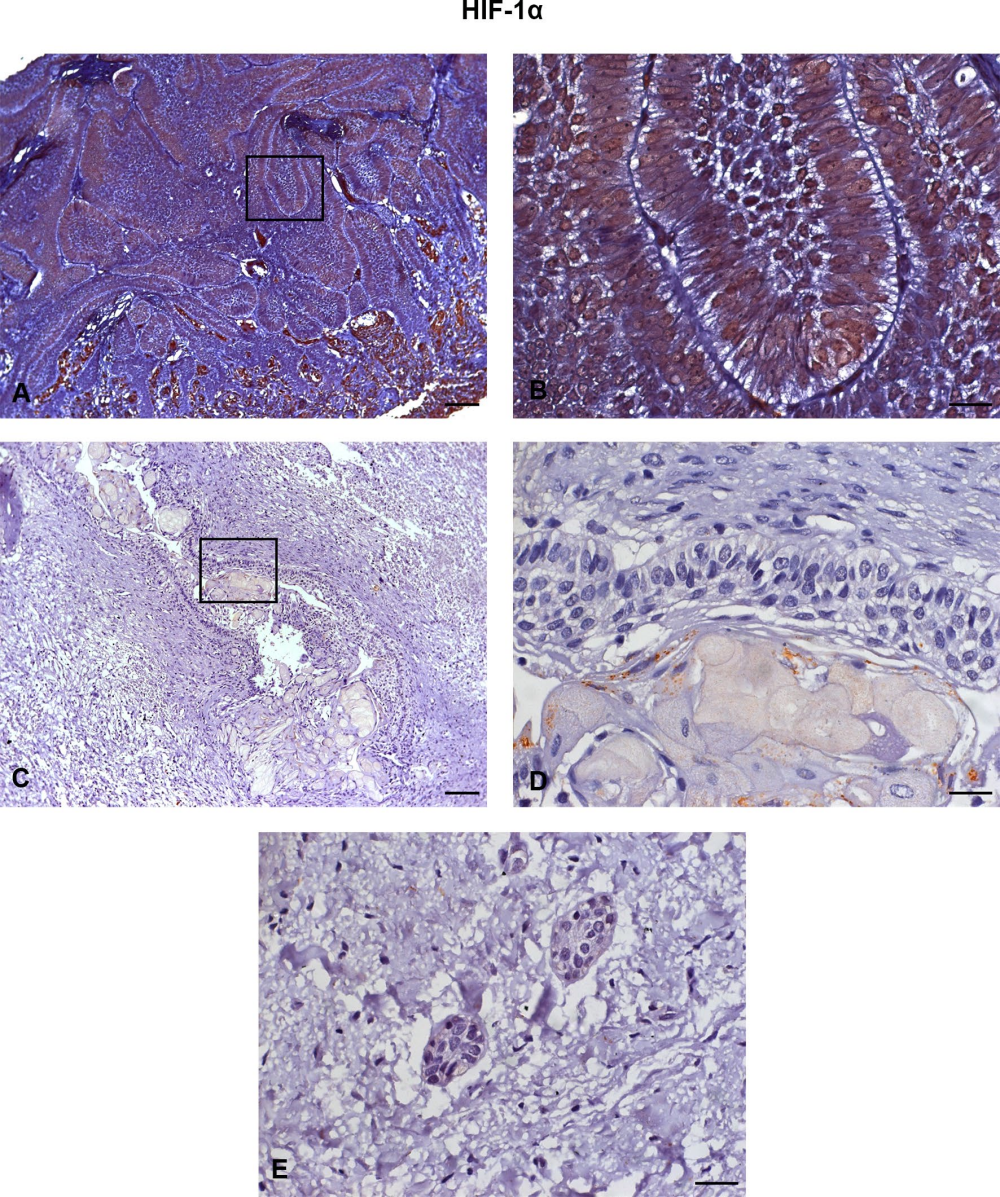
Immunostaining of hypoxia-inducible factor 1 alpha (HIF-1α) in ameloblastomas (AMEs), calcifying odontogenic cysts (COCs), and dental follicles (DFs). (**A**,**B**) Strong immunoexpression, predominantly in the nucleus of ameloblastoma tumour cells. (**C**,**D**) Low intensity immunoexpression in a calcifying odontogenic cyst. (**E**) Low immunoexpression in the dental follicle. Immunoperoxidase. Scale bar: (**A**,**C**) 100 µm; (**B**,**D**,**E**) 20 µm.

**Figure 2:**
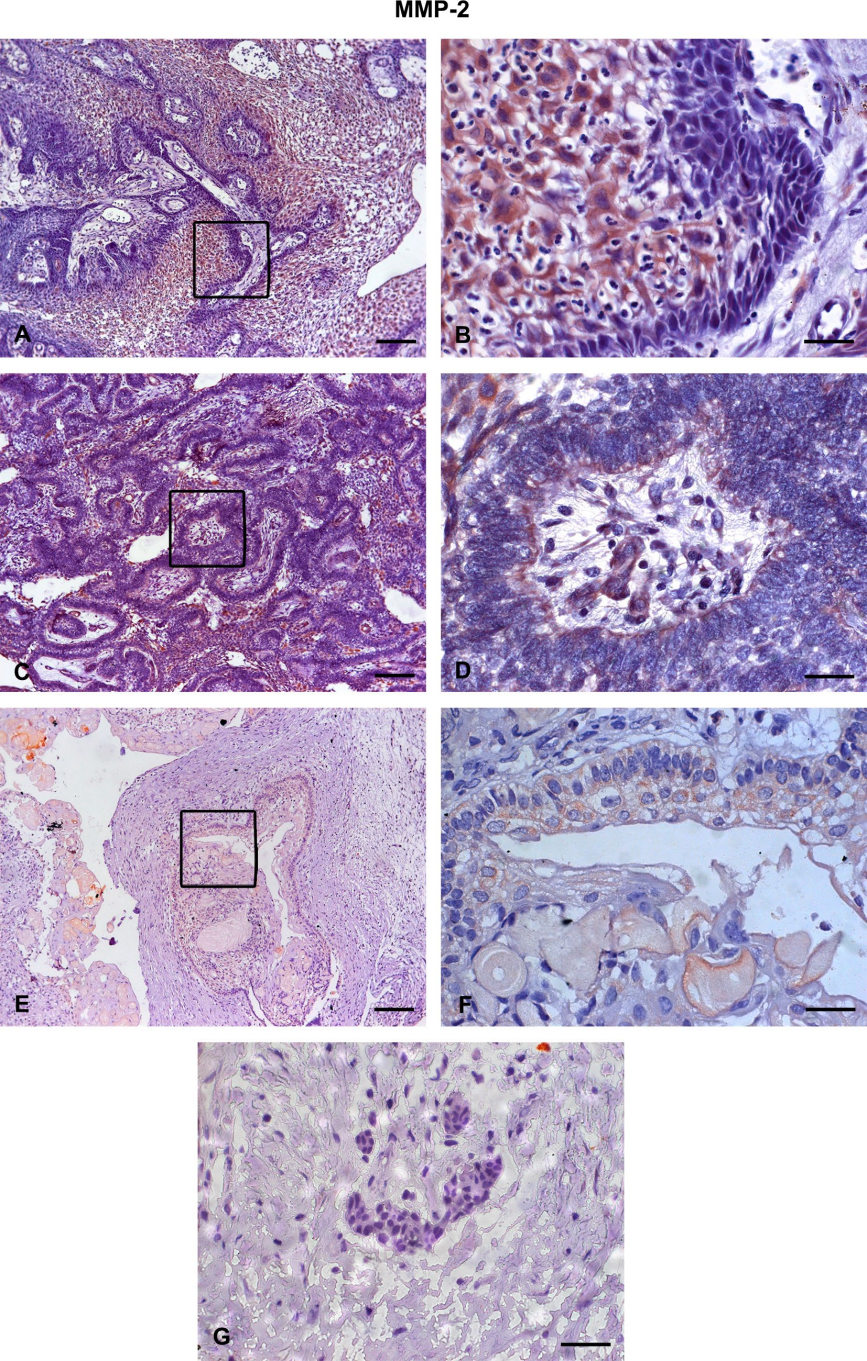
Immunostaining of matrix metalloproteinase-2 (MMP-2) in ameloblastomas (AMEs), calcifying odontogenic cysts (COCs), and dental follicles (DFs). (**A**,**B**) Strong cytoplasmic immunostaining in the central cells of the islands, formed by the ameloblastoma tumour epithelium. (**C**,**D**) Strong cytoplasmic immunostaining in the peripheral cells of the islands, formed by the ameloblastoma tumour epithelium. (**E**,**F**) Low intensity immunoexpression in a calcifying odontogenic cyst. (**G**) Low immunoexpression in a dental follicle. Immunoperoxidase. Scale bar: (**A**,**C**) 100 µm; (**B**,**D**,**E**) 20 µm.

**Figure 3:**
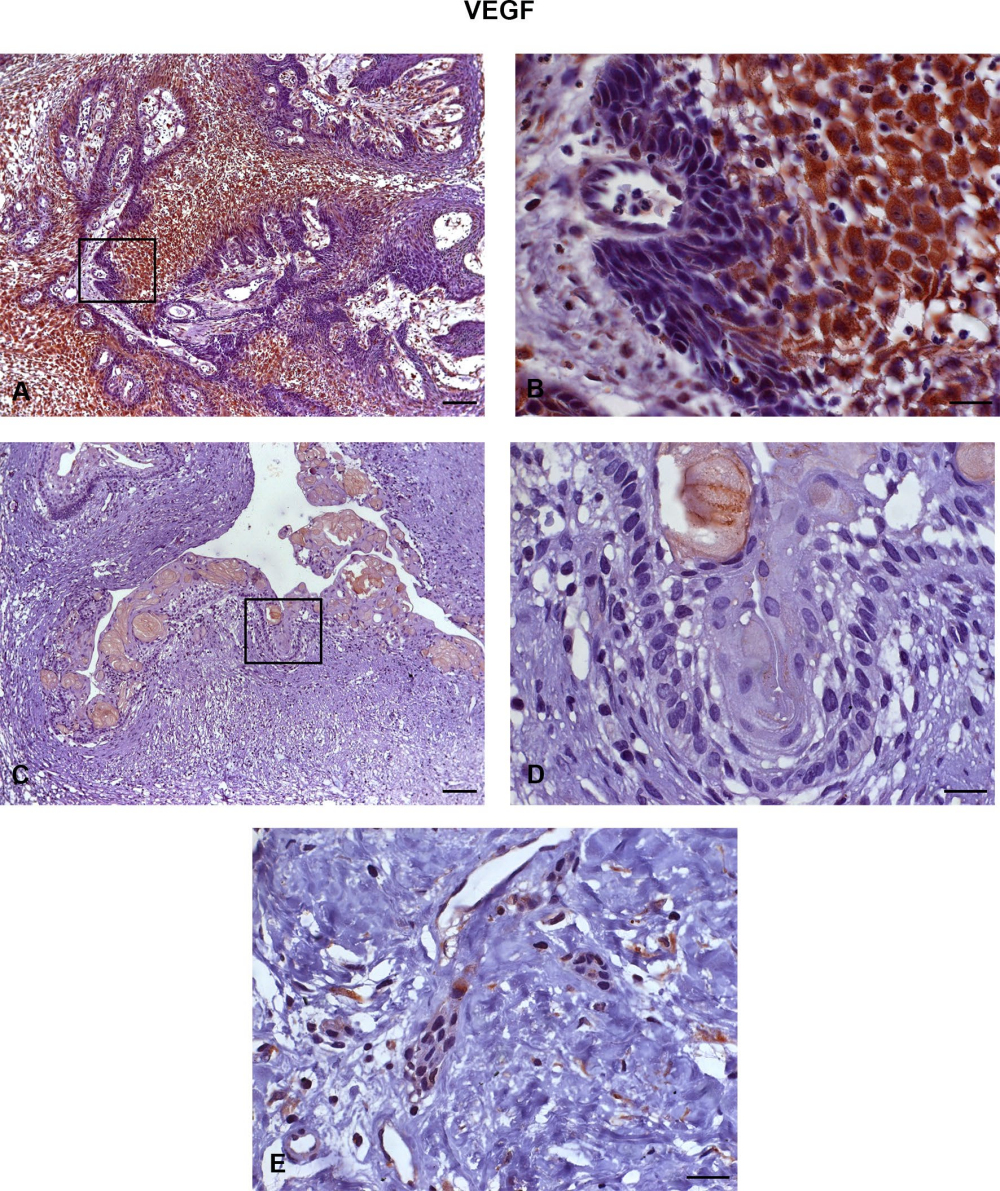
Immunostaining of the vascular endothelial growth factor (VEGF) in ameloblastomas (AMEs), calcifying odontogenic cysts (COCs), and dental follicles (DFs). (**A**,**B**) Strong immunostaining, predominantly cytoplasmic, in the central cells of the islands formed by the ameloblastoma tumour epithelium. (**C**,**D**) Low intensity immunoexpression in a calcifying odontogenic cyst. (**E**) Low immunoexpression in a dental follicle. Immunoperoxidase. Scale bar: (**A**,**C**,**E**) 100 µm; (**B**,**D**,**F**,**G**) 20 µm.

**Figure 4:**
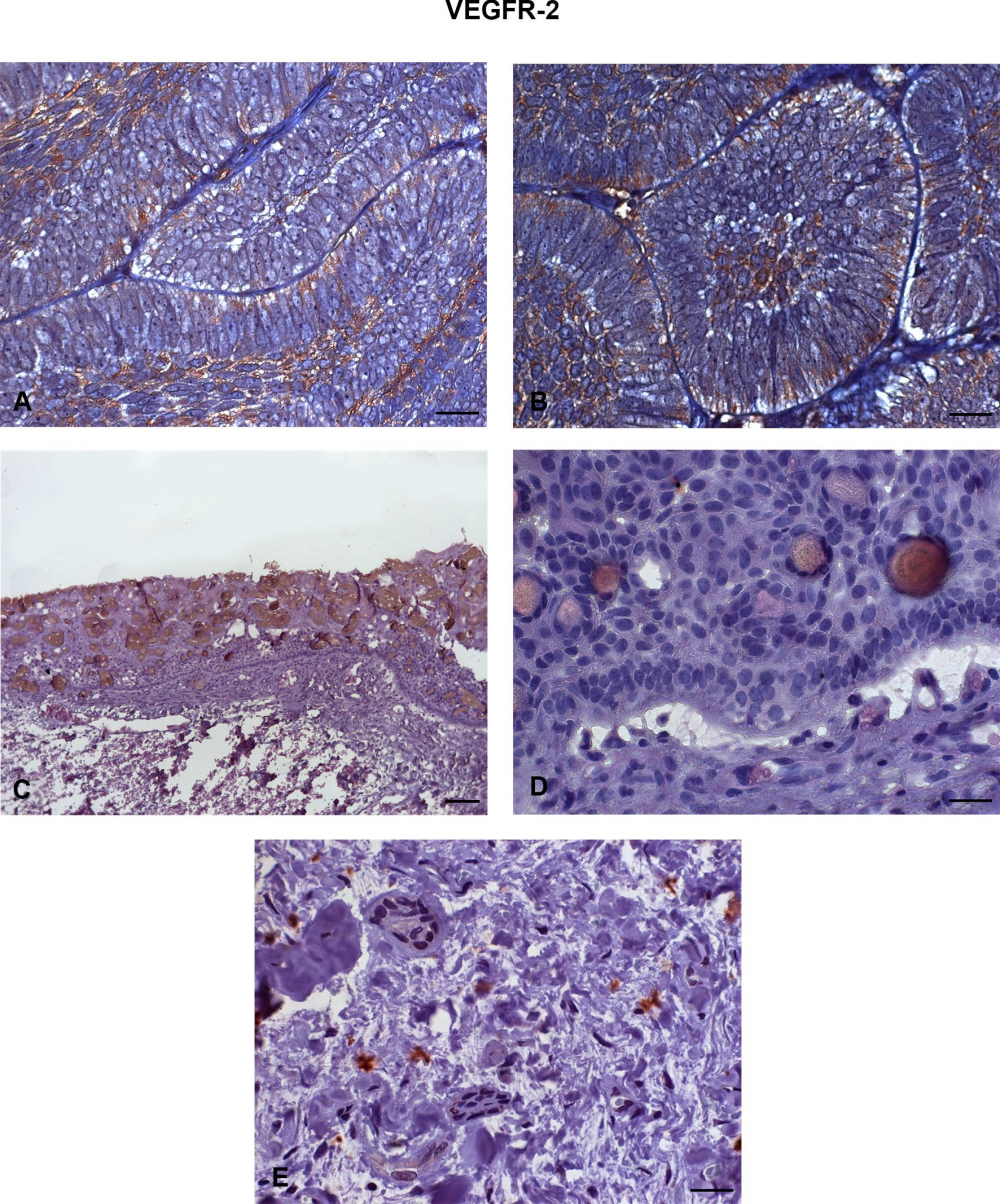
Immunostaining of the vascular endothelial growth factor receptor (VEGFR-2) in ameloblastomas (AMEs), calcifying odontogenic cysts (COCs), and dental follicles (DFs). (**A**,**B**) Strong immunostaining in the cell membrane of ameloblastoma tumour cells. (**C**,**D**) Low intensity immunoexpression in a calcifying odontogenic cyst. (**E**) Low immunoexpression in a dental follicle. Immunoperoxidase. Scale bar: (**A**,**B**,**D**,**E**) 20 µm; (**c**) 100 µm.

All AME samples expressed HIF-1α, MMP-2, VEGF/VEGFA, and VEGFR-2. There was a strong immunoexpression of HIF-1α, predominantly in the nucleus of tumour cells (Fig. 1A,B). Strong cytoplasmic immunostaining of MMP-2 was observed mostly in the central cells of the islands formed by the tumour epithelium (Fig. 2A,B) and in the peripheral cells of these islands (Fig. 2C,D). VEGF also showed strong immunoexpression in the central cells of the islands formed by the tumour epithelium, predominantly in the cytoplasm (Fig. 3A,B). VEGFR-2 showed immunostaining in the cell membrane of tumour cells (Fig. 4A,B). All proteins showed low immunoexpression in tumour stromal cells.

The COC samples showed low or no immunoexpression of HIF-1α (Fig. 1C,D), MMP-2 (Fig. 2E,F), VEGF/VEGFA (Fig. 3C,D), and VEGFR-2 (Fig. 4C,D). The DF samples showed low or no immunoexpression of HIF-1α (Fig. 1E), MMP-2 (Fig. 2G), VEGF/VEGFA (Fig. 3E), and VEGFR-2 (Fig. 4E).

**Figure 5:**
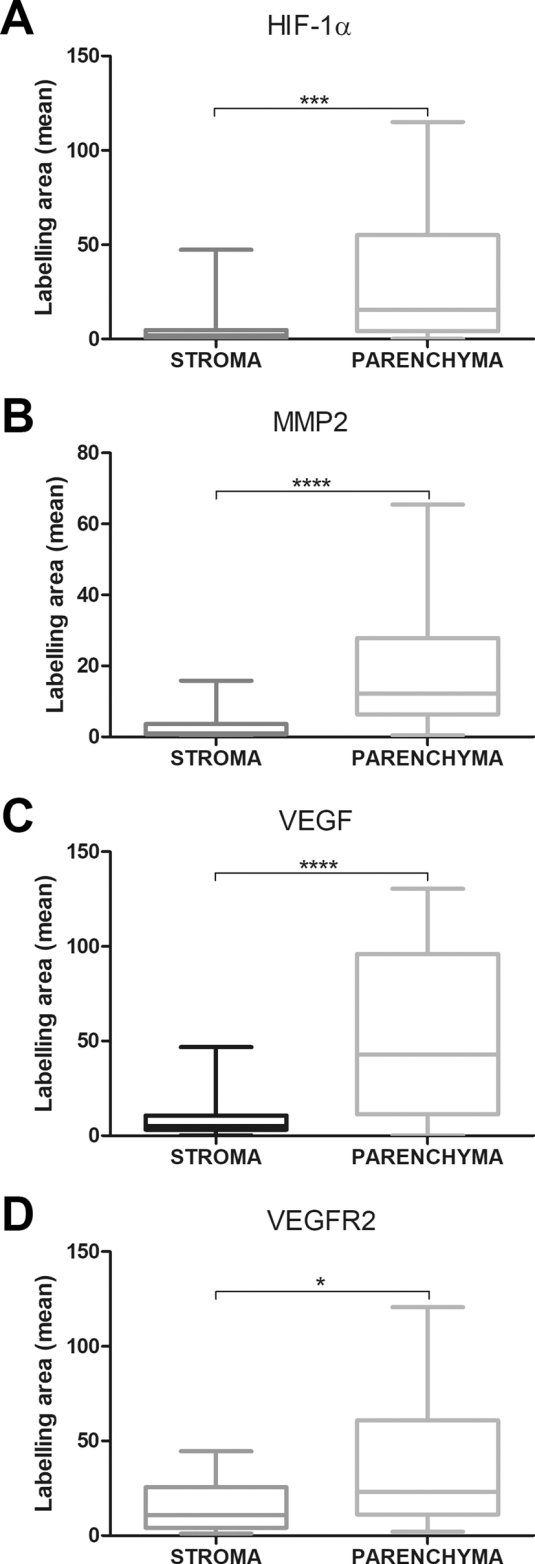
In ameloblastomas, the expression of (**A**) hypoxia-inducible factor 1 alpha (HIF-1α), (**B**) matrix metalloproteinase-2 (MMP-2), (**C**) vascular endothelial growth factor (VEGF), and (**D**) vascular endothelial growth factor receptor 2 (VEGFR-2) was higher in the tumour parenchyma than in the tumour stroma. *p < 0.5; **p < 0.1; ***p < 0.001.

There was a statistically significant difference in the immunoexpression of HIF-1α, MMP-2, VEGF/VEGFA, and VEGFR-2 proteins between the parenchyma of tumour cells and the stroma of tumour cells (Fig. 5A–D), where the parenchyma cells of AME showed a higher immunoexpression compared to the stromal cells.

**Figure 6:**
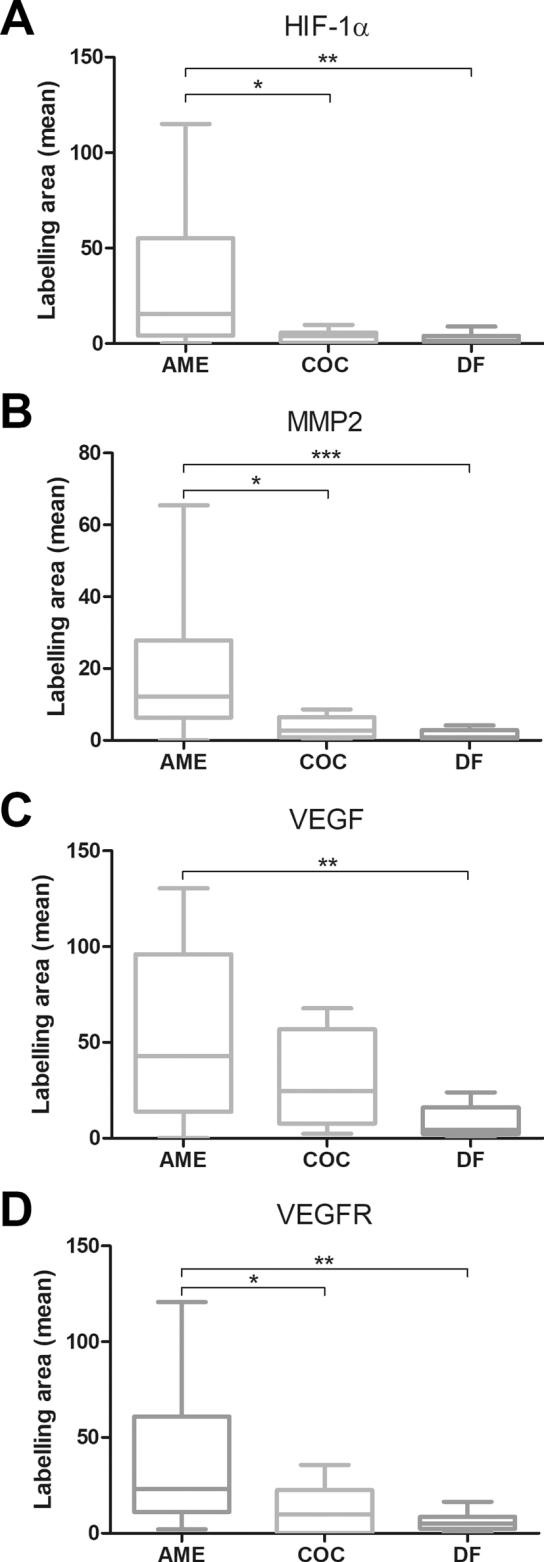
Comparison of (**A**) hypoxia-inducible factor 1 alpha (HIF-1α), (**B**)matrix metalloproteinase-2 (MMP-2), (**C**) vascular endothelial growth factor (VEGF), and (**D**) vascular endothelial growth factor receptor 2 (VEGFR-2) immunoexpression between ameloblastoma (AME), calcifying odontogenic cyst (COC) and dental follicle (DF) samples. *p < 0.5; **p < 0.1; ***p < 0.001.

It was also observed, significant difference in the immunoexpression of HIF-1α, VEGF/VEGFA, and VEGFR-2 between AME and COC samples and between AME and DF samples, where the expression of these proteins was higher in AME than in COC and DF (Fig. 6A,B,D), and there was a statistically significant difference in MMP-2 immunoexpression between AME and DF samples, where the expression was higher in AME than in COC and DF (Fig. 6C).

## Discussion

In our study, AME samples presented strong HIF-1α immunoexpression, predominantly in the nuclei of tumour parenchyma cells, where the transcription factor is active and triggers the transcription of several genes, initiating several mechanisms, such as angiogenesis, cell proliferation, and tumour invasion^4,5,34^. Solid tumours often contain regions with low oxygen concentrations around tumour cells, due to insufficient blood supply, resulting in intratumoral hypoxia^4,5^. Hypoxia-induced factor 1 (HIF-1) is a transcriptional activator responsible for the regulation of elements responsive to this phenomenon. In a hypoxic condition, HIF-1α binds with the HIF-1β subunit, becoming active and migrating to the cell nucleus in order to regulate cell survival mechanisms^35^, this corroborates with our findings, suggesting that the HIF-1α found was active. Its activation plays an essential role in the invasive process, being fundamental for tumour growth and aggressiveness, with overexpression observed in many human tumours^4,36–38^. Immunohistochemistry studies have observed the overexpression of HIF-1α in AME and have discovered that this is associated with its aggressive biological behaviour^17–19^.

Our results show a strong cytoplasmic immunostaining of MMP-2 in the central cells of the islands formed by the AME tumour epithelium, a region most likely to suffer intratumoral hypoxia. MMPs are secreted by stromal cells, endothelial cells, or by tumour cells themselves^10,39^. Pinheiro et al.^39^ also observed, through immunohistochemistry, the cytoplasmic expression of MMP-2 in the tumour parenchyma of AMEs and implied the high expression of this protein with the most aggressive infiltrative behaviour of this neoplasm.

ECM degradation by these MMPs stimulates the release of angiogenic and growth factors, such as VEGF, which is considered to be the most important inducer of angiogenesis and vascular permeability^9,10^. Hypoxia may also induce macrophages to produce more VEGF and suppress immune response^40^. Tumours induce neovascularization in order to acquire nutrients for continuous growth and metastatic spread. This “angiogenic switch” is induced by VEGF, which in turn can be produced by cancer cells and host stromal cells in a tumour^8,41^, which contributes to the disruption of the balance between angiogenic promoters and inhibitors^42^. Experimental and clinical reports have confirmed that VEGF plays a central role in regulating angiogenesis and vasculogenesis in solid tumours^43,44^. Its signalling may affect several significant tumour functions in addition to vascular permeability and neovascularization^45^, such as tumour cell proliferation, migration, and autocrine invasion^46,47^. Kumamoto et al.^20^ and Dineshkumar et al.^21^ demonstrated a strong cytoplasmic expression of VEGF in AME, however, the immunoexpression was concentrated in the peripheral cells of the tumour epithelium, indicating the proangiogenic role of this protein.

In our study, VEGF showed strong cytoplasmic immunoexpression in the central cells of the islands formed by the tumour parenchyma, farthest region from the stroma, the supporting tissue, indicating the possible activity of this protein in intratumoral hypoxia area, suggesting an alternative role to its angiogenic function. Tong et al.^45^ suggested that the VEGF expression in a head and neck carcinoma cell line may play two different roles in tumorigenesis: (1) through its paracrine function, essential for tumour-associated angiogenesis, and (2) through its autocrine function, where VEGF plays an important role by directly enhancing mitogenesis and invasiveness by maintaining proliferation, enhancing survival, and increasing the invasion of carcinomas. Therefore, VEGF may serve a proangiogenic and protumorigenic role in the pathogenesis of neoplasms. In addition, Tong et al.^45^ stated that VEGF-targeted therapy has the potential to fulfil both anti-angiogenic and anti-tumorigenic functions. Higher VEGF expression in tumour parenchyma, compared to tumour stroma, suggests a pro-tumorigenic role instead of a pro-angiogenic role, where it would be possible to observe a higher expression of VEGF in the stromal microenvironment. This hypothesis has been reinforced by several authors^8,45–47^.

Cystic lesions showed an increased concentration of VEGF in the cystic fluid that was produced by parenchymal cells, which can induce proliferation in cyst lining epithelial cells^6,27,28^. Altered Wnt pathway signalling has been identified in COCs^35^ as well as ameloblastomas^36^, and may be one of the factors involved in expression of VEGF in these lesions^22^. In the study by Dineshkumar et al.^21^, there was no statistically significant difference in VEGF expression between AME and COC. In our study, VEGF had a higher expression in AME than in COC; however, there was no statistical difference. This finding reinforces the fact that COC, despite being a cystic lesion of odontogenic origin and having a milder clinical behaviour when compared to AME, still has aggressive characteristics^1^.

Among VEGF receptors (VEGFRs), VEGFR-2 is overexpressed under hypoxia^15^. In our study, VEGFR-2 immunoexpression was observed in the cell membrane of tumour cells. VEGFR-2, localized on endothelial cell surfaces, appears to regulate VEGF endothelial cell permeability and proliferation^11,48^. VEGF receptors were originally believed to be expressed only in endothelial cells, however, it was later shown that they could also be expressed in tumour cells, including head and neck cancers^45,49^. This is the first study to evaluate the expression of this receptor in AME and COC.

Our results show higher immunoexpression of HIF-1α, MMP-2, VEGF, and VEGFR-2 in ameloblastomas when compared to calcifying odontogenic cysts and dental follicles, suggesting that the relationship between these proteins may contribute to the behaviour of this neoplasm. As the centre of the tumour islands showing higher expression of HIF-1α curiously promote higher VEGF expression in the same region, as well as a higher expression of VEGFR-2 in tumour cells. From this, we can extrapolate that VEGF in the central cells of islands formed by tumour parenchyma may signal peripheral cells for greater proliferation and invasion, which is likely to be mediated by MMP-2 expression. The methods used in this work, although indicating the expression of the studied proteins, limit the depth of our knowledge regarding the role of HIF-1α, MMP-2, VEGF, and VEGFR-2, as the expression of proangiogenic proteins in the centre of tumour epithelial islands suggest a secondary role of these proteins in the proliferation, survival, and invasion of ameloblastoma. In this sense, mechanistic studies must be done to answer these questions.

## Methods

### Ethical approval

The Human Research Ethics Committee of the Health Sciences Institute of the Federal University of Pará approved this research (protocol number: 2371410). All procedures performed were in accordance with the ethical standards of the national research committee and with the 1964 Helsinki declaration and its later amendments or comparable ethical standards. In addition, informed consent was obtained from all participants and/or their legal guardians.

### Samples

Twenty four AME samples were retrieved from the files of the Department of Oral Pathology, School of Dentistry, Universidade Federal do Pará (UFPA), Belem, PA, Brazil, and ten COC and nine dental follicle (DF) samples were retrieved from the files of the Department of Clinic, Pathology, and Dental Surgery, Universidade Federal de Minas Gerais (UFMG), Belo Horizonte, Minas Gerais, Brazil.

### Immunohistochemistry

Immunostaining was performed using an immunoperoxidase assay and the EnVision technique as previously described by Costa et al.^35^. Formalin-fixed, paraffin-embedded tissues were studied by immunohistochemistry. Sections of a 5-µm thickness were obtained and mounted on 3-aminopropyltriethoxysilane-coated slides (SIGMA-ALDRICH). The sections were deparaffinised in xylol and hydrated in graded ethanol solutions. The sections were immersed in 20% H_2_O_2_ and methanol in a 1:1 ratio for 20 min to inhibit endogenous peroxidase activity. A citrate buffer (pH 6.0) was used for antigen retrieval in a Pascal chamber (DAKO) after immersion for 30 s. Subsequently, non-specific biding sites were blocked with 1% bovine serum albumin (BSA, SIGMA-ALDRICH) in a phosphate-buffered saline (PBS) solution for 1 h. The slides were incubated with the respective primary antibodies against HIF-1α (1:100, MILLIPORE Cat# MAB5382, RRID:AB_177967), MMP-2 (1:100, ST. JOHN’S LABORATORY, Cat# STJ94163, RRID:AB_2819762), VEGF/VEGFA (1:100, THERMO FISHER SCIENTIFIC Cat# PA5-16754, RRID:AB_10979267) and VEGFR-2 (1:100, THERMO FISHER SCIENTIFIC Cat# PA5-16487, RRID:AB_10978670). All primary antibodies were diluted in PBS and incubated for 1 h at room temperature. Afterwards, the sections were incubated for 30 with EnVision Plus (DAKO) detection system (DAKO). Diaminobenzidine (DAKO) was used as the chromogen, and sections were counterstained with Mayer’s haematoxylin (SIGMA-ALDRICH). For negative controls, primary antibodies were interchanged with non-immune serum.

### Immunostaining evaluation

To assess the intensity of HIF-1α, MMP-2, VEGF, and VEGFR-2 staining, brightfield images from at least five randomly selected fields from each sample were acquired using an AxioScope microscope (CARL ZEISS) equipped with a CCD colour camera (AxiocCam HRc; CARL ZEISS). The images were acquired at the same magnification (40×). Areas of diaminobenzidine staining were analysed using the immunohistochemistry (IHC) image analysis toolbox plugin (written by Jie Shu, Guoping Qiu and Mohammad Ilyas, https://imagej.nih.gov/ij/plugins/ihc-toolbox/index.html) of ImageJ (public domain software developed by Wayne Rasband; NIMH, NIH, Bethesda, MD, USA, https://rsbweb.nih.gov/ij). The evaluation of immunostaining was performed by measuring the mean grey value, which consists of the average grey value within the selection. Both neoplastic cells and stromal cells were included in the quantification.

### Statistical analysis

The data were analysed using the GraphPad Prism 8 software (Graph Pad Software, Inc., San Diego, CA, USA). A Mann–Whitney test was carried out to evaluate the statistical differences in protein immunoexpression between the stroma and the tumour parenchyma (95% confidence interval). The Kruskal–Wallis test and Dunn’s post-test were carried out to evaluate the statistical differences in protein immunoexpression between AME, COC, and DF.
